# Sports Drinks for Rehydration, Amelioration of Fatigue, and Recovery from Exertion

**DOI:** 10.3390/nu18111687

**Published:** 2026-05-25

**Authors:** Katsuhiko Suzuki

**Affiliations:** Faculty of Sport Sciences, Waseda University, Tokorozawa 359-1192, Saitama, Japan; katsu.suzu@waseda.jp; Tel.: +81-4-2947-6898; Fax: +81-4-2947-6801

**Keywords:** exercise, dehydration, fluid ingestion, absorption, osmolarity, immunodepression, inflammation

## Abstract

Sports drinks have traditionally been formulated as carbohydrate–electrolyte beverages to support fluid replacement and energy provision during exercise. However, commercially available and experimentally tested formulations now include amino acids, proteins, phytochemicals, caffeine, menthol, ketone-related nutrients, and other functional ingredients intended to support thermoregulation, performance, or post-exercise recovery. This narrative review summarizes evidence on sports drinks and related functional beverages, with emphasis on hydration, gastric emptying and intestinal absorption, thermoregulation, biomarkers of hydration and recovery, and potential effects beyond hydration, including fatigue, muscle and organ damage, inflammation, and immune responses. Because available studies vary widely in population, exercise mode, environmental conditions, beverage composition, intake timing, and outcome measures, the evidence should be interpreted cautiously. A functional distinction is made between hydration-oriented carbohydrate–electrolyte beverages and beverages primarily designed for ergogenic or recovery-oriented purposes. Current evidence supports the practical value of appropriate fluid, electrolyte, and carbohydrate intake for maintaining hydration and exercise performance, whereas evidence for broader effects on inflammation, immunodepression, organ protection, and recovery remains context-dependent. Future studies should differentiate acute responses from longer-term adaptations, consider population-specific needs, and use standardized, context-appropriate biomarkers and ecologically valid protocols.

## 1. Key Points

1. Sports drinks are liquids that efficiently replenish the water, sodium and other electrolytes lost from the body because of sweating during prolonged exercise and that also contain carbohydrates as a source of energy for exercise.
2. Beverages with a variety of ingredients and characteristics have emerged, and their uses have also become more widely diversified. However, from the perspective of consuming liquids during and after exercise, sports drinks are defined as beverages that ameliorate dehydration, fatigue, and other physical distemperature caused by exercise, and that maintain, or improve performance or accelerate recovery.
3. The effectiveness of the beverages differs depending on conditions such as composition, amount and timing of intake, and other intrinsic and external factors.

## 2. Background

**Figure 1:**
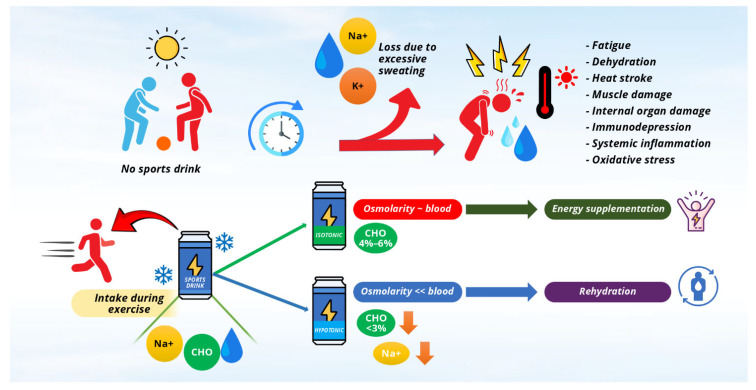
Sports drinks for rehydration and amelioration of exercise-induced adverse risks.

Water is a fundamental component of the human body (~70% of body composition) and plays a crucial role in numerous physiological processes [1,2,3]. Body water and electrolytes are lost in sweat during exercise [4,5,6,7,8]. Following long-duration exercise, glycogen stores in muscle and liver are also depleted, and blood glucose decreases [9,10,11,12]. Under those conditions, fluid intake can attenuate the adverse effects of dehydration on the body temperature and homeostasis as shown in Figure 1 [4,5,6,7,8]. Exercise performance improves with the ingestion of sports drinks containing carbohydrates because their consumption delays the onset of fatigue by sparing muscle glycogen and maintaining blood glucose [4,5,6,13,14,15]. Beverages with added metabolic stimulants and medications such as caffeine are commercially available as ergogenic aids to enhance exercise performance [16,17,18,19,20,21,22]. Beverages with added functional nutritive ingredients such as amino acids and phytochemicals can ameliorate muscle damage, fatigue, inflammation, immunodepression, and other harmful effects caused by exertion [23,24,25,26,27]. Here, the features, usefulness, and problems of sports drinks are reviewed.

This article is intended as a narrative and integrative review rather than a systematic review. Given the relatively limited number of studies and the substantial heterogeneity in study designs, populations, interventions, and outcome measures related to sports drinks and functional beverages, a formal systematic review with quantitative synthesis was considered inappropriate at this stage. Searches were conducted using combinations of terms including “sports drink,” “carbohydrate–electrolyte beverage,” “rehydration,” “exercise,” “thermoregulation,” “gastric emptying,” “recovery,” “inflammation,” “immune function,” “fatigue,” “amino acids,” “caffeine,” “ketone,” and “functional beverage.” Priority was given to peer-reviewed experimental studies, systematic reviews, position statements, and studies directly examining beverage ingestion in relation to exercise, hydration, thermoregulation, performance, or recovery. Because the review was narrative in scope, no formal risk-of-bias assessment or quantitative synthesis was performed. The findings should therefore be interpreted not as definitive or broadly generalizable conclusions, but rather as indications of emerging trends and ongoing areas of research.

## 3. Definition of “Sports Drink”

During prolonged exercise, performance declines because of dehydration and electrolyte loss caused by sweating, glycogen depletion in skeletal muscles and liver, and decreased blood glucose [4,5,9,12]. Replenishment not only with water containing electrolytes, but also with energy substrates, is therefore necessary, and the intake of a sports drink is extremely useful as a means of quickly replacing those lost components [4,5,6,8].

The 1998 Muraoka’s review of sports drinks summarized the scientific basis for their effects and effective supplementation methods using the definition “sports drinks are solutions containing carbohydrates and electrolytes” [7]. Since then, no other definition of sports drinks has emerged, but defining “sports drink” has become ever more difficult because multiple beverages with a variety of ingredients and characteristics have emerged since the start of the 2000s, and the uses of those beverages have also become more widely diversified [6,23,28,29]. Even when considered from the perspective of peri-exercise fluid ingestion, sports drinks can be broadly defined as beverages specifically formulated to ameliorate dehydration, fatigue, and exercise-associated physiological disturbances, while maintaining or improving performance or accelerating recovery through fluid replacement, electrolyte replenishment, and/or substrate provision. In this review, a functional classification framework based on beverage composition and intended use is adopted to organize and contextualize the wide range of drinks discussed. Beverages are broadly classified into (i) carbohydrate–electrolyte drinks primarily intended for hydration and (ii) beverages not primarily designed for hydration. The latter category includes products aimed at enhancing exercise performance or providing ergogenic effects, as well as beverages containing functional nutrients that are primarily intended to support post-exercise recovery and conditioning. While evidence from traditional reviews of conventional sports drinks is also comprehensively referenced, this review places particular emphasis on recent research trends concerning emerging sports drinks that target outcomes beyond hydration alone.

To improve interpretability, the evidence discussed in this review is considered according to three contextual dimensions: timing of intake and response, namely acute responses during or immediately after exercise versus longer-term adaptations; the primary intended outcome, namely hydration, thermoregulation, performance, or recovery; and the target population or exercise context. This framework is particularly important because the same beverage component may have different implications depending on whether it is used during dehydration, during euhydrated exercise, or during post-exercise recovery.

## 4. Types and Characteristics of Basic/Traditional Sports Drinks

The wider array of beverages will be introduced later, but the traditional carbohydrate electrolyte-based sports drinks are basically classified into isotonic or hypotonic depending on differences in their osmolarity [8,30,31,32,33,34,35]. As shown in Figure 1, isotonic drinks are excellent for energy supplementation during exercise because their osmolarity closely resembles extracellular fluids such as blood, allowing their water, sugar, and salt to be absorbed in better balance [30,36,37,38,39]. These beverages have a relatively high carbohydrate content of about 4–6%, and most sports drinks on the market today are isotonic drinks [40,41,42].

In contrast, hypotonic drinks have a lower concentration of sodium and carbohydrates and a lower osmolarity than that of extracellular fluid [8,30,33,43,44,45]. These beverages typically contain less than 3% carbohydrates. They are particularly well suited for rehydration during exercise, because their water is absorbed quickly in the intestinal tract under conditions of fluid loss due to exercise-induced sweating [8,30,33,46].

Oral rehydration solutions have a carbohydrate content similar to that of hypotonic drinks and are superior in terms of rehydration; however, they contain more sodium than sports drinks, making them more suitable for early recovery from dehydration caused by vomiting, diarrhea, fever, or profuse sweating caused by infectious conditions such as gastroenteritis [47,48,49,50]. Meanwhile, a recent study showed reduced excretion of urinary creatinine and uric acid following exertion, suggesting decreased waste product elimination from the body during recovery [33].

## 5. Mechanisms of Water Absorption

Because little to no fluid is absorbed in the stomach and nearly all fluid absorption occurs in the small intestine, the speed with which an orally ingested sports drink is absorbed into the body depends primarily on the gastric emptying rate (GER) [4,5,50]. The GER is influenced by a variety of factors, but the intensity of exercise can especially affect absorption by decreasing splanchnic perfusion [4,51,52,53,54,55,56]. With respect to sports drinks consuming during exercise, although there are osmolality-dependent effects in addition to volume-dependent effects on gastric emptying, the GER for hyperosmotic solutions of 500 mOsm/L or more is slower than that for water [57]. On the other hand, ingestion of beverages warmer than body temperature increases the GER [58], improving appetite loss after exercise, and has been reported to have a positive effect on nutritional intake and absorption [59,60,61,62,63,64,65]. In any case, faster recovery from dehydration and muscle damage/fatigue after the completion of exercise requires supplementation not just with fluid, but also with nutrients such as carbohydrates, proteins, and their component amino acids [66]. In terms of the GER, warm drinks containing such nutrients may be more helpful than cooled beverages which can decrease gastrointestinal functioning during recovery and/or at rest [58]. It should be noted, however, that evidence for temperature effects on gastric emptying and subsequent recovery has been derived primarily from controlled laboratory conditions, and caution is warranted when translating these findings into practical field recommendations. Furthermore, the relevance of GER should be interpreted in the context of timing: during ongoing exercise, rapid fluid absorption is the primary objective, whereas during the post-exercise recovery phase, provision of carbohydrates, proteins, and amino acids alongside fluid becomes increasingly important for restoring muscle glycogen and supporting tissue repair [66].

In the small intestine, co-transporters of sodium and glucose are present on the luminal side of the mucosal epithelium, resulting in sodium being taken up into the body together with glucose; water is also passively absorbed along the osmolarity gradient created [5,67,68]. The co-ingestion of sodium and glucose in sports drinks therefore not only supplements salt loss from sweating and provides a source of energy for exercise but also helps the body absorb water more quickly [5,67,68,69,70,71,72,73,74].

The ratio of electrolytes to sugar in hypotonic drinks is designed such that the co-transport mechanism tends toward the most effective water absorption [8]; however, oral rehydration solutions, with their high concentration of sodium tend toward an even more optimum ratio [47,50]. In the intestinal tract, fluids are adjusted to achieve the same osmolarity as the extracellular fluid before water is absorbed, regardless of the composition of the solution ingested [5,75]. However, beverages with a high sugar content increase osmolarity in the intestinal tract and can cause diarrhea [76] as well as a rise in blood glucose that can induce adverse effects as will be explained in the next subsection. Beverages with an osmolarity not exceeding isotonicity are therefore recommended [8,77].

Recently, sugar-free hydration drinks have also been gaining attention. Amino acid beverages have been reported to promote water absorption. In addition to gut Na^+^-dependent glucose transporters, Na^+^-dependent amino acid (AA) gut transporters [78] also facilitate water and electrolyte absorption comparably to glucose [79]. Several papers have reported its effectiveness [80,81,82].

## 6. Appropriate Method of Hydration During Exercise and the Harmful Effects of Overconsumption

An effective method of hydration is to keep a beverage close at hand during exercise and work, and to take frequent small sips before thirst sets in [83]. This approach attenuates the adverse effects of dehydration on physical activity and health, but fluid needs are notably individualistic, relying on factors such as personal sweat rate, exercise mode, intensity, and duration together with environmental conditions [4,83]. Furthermore, characteristics and rules unique to each sporting environment, the event uniform and equipment, and the availability of fluid during both training and competition can greatly influence whether hydration can be optimized during activity [4,83]. In addition, a study evaluating dehydration in athletes reported that 66.7% of athletes were dehydrated even before exercise, indicating that hydration before training is also important [84].

The timing of beverage intake is another factor requiring careful consideration [85]. If a large amount of sugar is ingested immediately before the start of exercise, blood glucose rises and insulin is secreted; exercise performed in this situation can easily lead to hypoglycemia [86]. This potential for insulin shock requires attention, because impaired consciousness can result [86,87]. Furthermore, the large amounts of sugar and citric acid that some beverages contain to relieve fatigue can contribute to diabetes, obesity, and dental caries when consumed in excess [76,88].

Insulin is also secreted when a large amount of carbohydrate is ingested in the absence of sport activity, and with unnecessary repetition of such consumption, the capacity for insulin secretion declines and the blood glucose rises. The body’s subsequent attempts to dilute blood sugar causes thirst, and fluid intake increases. If beverages high in carbohydrate, such as sports drinks, are consumed again, a vicious cycle ensues. This diabetic condition, called plastic bottle syndrome or PET bottle syndrome (soft-drink ketosis or hyperosmolar hyperglycemic syndrome), can lead to impaired consciousness and even death, and frequent consumption of sports drinks instead of water in the non-exercise setting should be avoided [86,87]. In addition, there are concerns on the effects of excessive consumption on overweight [89]. Sports drinks, as the name suggests, are intended for supplementing the losses from sweating and energy expenditure during sports. At other times, such beverages should be consumed in an amount commensurate with the related carbohydrate and electrolyte (salt) contents, considering the outside temperature and the consumer’s physical condition at the time. Although this is beyond the context of this review, another review article presents practical hydration solutions for such situations [4].

## 7. Evaluating Dehydration and Hydration

The sensation of thirst is a centrally mediated response to a deficit of body water, which is useful in spurring fluid intake; however, thirst is relatively insensitive in acutely tracking hydration status during exercise [4]. The dryness of the oral cavity, checked at the time of examination, can act as a clinical finding of dehydration. Recently, a device to evaluate dehydration by the degree of wetting of the oral cavity has been developed, and its widespread use may be useful in prevent dehydration and heat stroke [90]. On the other hand, it is well known that relatively severe thermal dehydration by a large amount of sweat loss is not completely recovered during heat exposure despite free access to water [91]. This is called “voluntary dehydration” and requires attention.

In previous studies of sports drinks, participants completed endurance exercises, and dehydration was evaluated based on weight loss attributable to sweating [92,93,94,95,96]. For example, in a study on fluid intake and the biologic response to cycling in a hot environment, pure water, isotonic drinks, and hypotonic drinks were each freely consumed on different days. Analysis revealed that hypotonic drinks, which have an osmotic pressure lower than that of body fluid, were easier for athletes to consume during exercise and ameliorated dehydration as evaluated by body weight change after exercise [8]. Thus, it became clear that the absorbability of beverages and their ability to improve dehydration and physical condition differ depending on their osmolarity and type of carbohydrate content [8].

## 8. Useful Urinary Indicators

In academic research, dehydration and hydration analyses sometimes use blood testing [3,5,8], but in sports settings, urine testing is more common [4,23,37,47]. Urine specific gravity or, strictly speaking, osmolarity is generally used as a typical test index for evaluating dehydration [97,98]. In these tests, highly concentrated urine indicates dehydration, whereas low value indicates that excess water is being excreted, making the urine lighter in color. Also, some test papers for urinalysis can detect urinary protein, occult blood, sugar, ketones, white blood cells, and even bacteria [99,100,101,102]. Those measures are useful because they can screen for the influences of nutrition, supplements, and diseases in addition to liquid intake and output [47,96]. Furthermore, there are some biomarkers for quantitative analyses in evaluating exercise-induced disorders. For example, in case of an all-out time trial of 3000 m running, inflammation-related markers including cytokines, chemokines, complements, and leukocyte activation markers were increased in urine, reflecting systemic inflammation [47,98,99,100,101,102,103,104,105,106,107]. Also, intestine- and liver-type fatty acid-binding proteins (I-FABP and L-FABP, respectively) increased, indicating internal organ damage whereas a muscle damage marker, titin N-fragments and oxidative stress markers did not change significantly following such a short-time intensive exercise [33,47,98,108,109,110,111,112,113,114,115,116,117,118].

It is important to note that the suitability of biomarkers for evaluating beverage efficacy depends strongly on the assessment context. Among the available indicators of hydration status, changes in body mass represent the most practical and accessible field-based marker. Body mass loss can be measured non-invasively and in near-real time without laboratory equipment, making it the most widely adopted indicator in applied sports settings. Current international consensus guidelines recommend that body mass loss during exercise be kept below 2% as a primary threshold for dehydration risk [83,92]. Urine osmolality and urine specific gravity provide complementary information and are feasible when portable measurement equipment is available on-site. Plasma osmolality and blood sodium concentration are more sensitive laboratory-based reference standards, but their applicability is limited outside controlled research environments. In contrast, markers such as creatine kinase, inflammatory cytokines, organ-damage-related proteins (e.g., I-FABP, L-FABP), and performance recovery indices are more relevant for assessing post-exercise recovery or longer-term adaptation-related outcomes, although their sensitivity and ecological applicability vary considerably across study designs. Accordingly, no single biomarker is universally optimal; outcome measures should be selected in accordance with the specific physiological domain, time course, and practical feasibility of the assessment context under investigation.

## 9. The Influence of Sports Drinks on Thermoregulation Following Exercise

**Figure 2:**
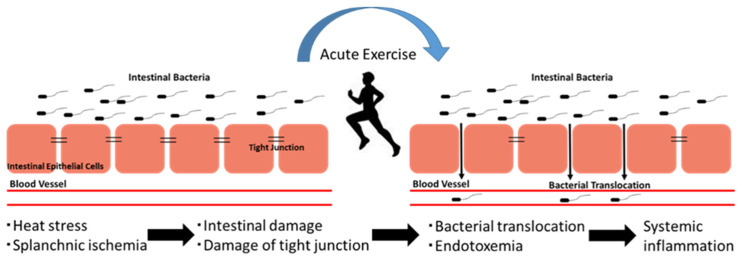
The concept of exercise-induced endotoxemia and systemic inflammation. Exercise induces intestinal barrier dysfunction and hyperpermeability. Subsequently, gut-derived bacteria translocate to the circulation and induce systemic inflammation [124].

During exercise, thermoregulatory mechanisms such as cardiovascular drift, skin blood flow, and sweat rate changes, but the working muscles produce heat, a process that is especially exacerbated in hot and humid environments; if heat dissipation by sweating cannot keep up, body temperature rises, exercise performance declines, and heat stroke can occur [4,119,120,121]. High-intensity or long-duration exercise can lead to ischemia of the internal organs because of redistribution of blood flow; strenuous exercise increases blood flow to skeletal muscle up to 100-fold, but deceases into the liver, kidney and testes [52,122,123,124]. In particular, the mucosal epithelium of the intestinal tract can be injured as shown in Figure 2, and if intestinal bacteria invade the bloodstream, endotoxemia (sepsis) can result, with inflammatory substances such as endotoxin (a bacterial toxin), and cytokines being spread throughout the body via the blood circulation (systemic inflammation) [124,125,126]. Heat stroke is a dangerous condition that can cause multiple organ failure and even death; examples of the effects include weakness and convulsions (skeletal muscle), acute renal failure and rhabdomyolysis (kidneys), and impaired consciousness (brain) [4,125,126,127].

The consumption of cooled beverages is effective in ameliorating dehydration and body temperature elevation [128,129]. In recent years, ice slurries have attracted attention as a new strategy of body cooling [128,129,130,131,132,133,134,135]. These sherbet-like beverages containing fine ice grains can simultaneously cool the body and supply carbohydrates, electrolytes, and other substances [134,135]. For example, a study found that ice slurries ingested before exercise were effective in reducing the increase in core body temperature expected during exercise loading in a hot environment; however, they showed no consistent trend in their effects on exercise performance or sweating [134]. On the other hand, in both men and women, consumption of an ice slurry after exercise in the heat was effective in lowering a core body temperature, that had risen after exercise, with men tending to sweat less and women experiencing blood pressure reductions after exercise; however, the mechanisms involved have not been elucidated because of large individual differences [135].

Cooling strategies employed for thermoregulation may serve distinct purposes depending on the timing and context of application. Cooling implemented before or during exercise is primarily intended to attenuate thermal strain and support performance under heat stress, whereas post-exercise cooling strategies are more commonly applied to facilitate the reduction in elevated body temperature and to support recovery processes. These differing objectives should be taken into account when interpreting the efficacy of cooling interventions.

Furthermore, consuming large amounts of ice-cold beverages may induce gastrointestinal disorders, nausea, and/or physical discomfort such as “brain freeze”/headaches. Therefore, it is recommended to implement internal cooling strategies during training sessions and simulated competitions and determine the optimal amount and temperature of beverages that individual athletes can tolerate [136]. Although several studies have reported beneficial effects of cooling strategies on thermoregulation, their influence on exercise performance and recovery outcomes remains inconsistent. These discrepancies likely reflect differences in cooling modality, timing of application, exercise intensity, environmental conditions, and individual responses. Accordingly, the reported effects should be interpreted cautiously and within the specific context in which each intervention is applied.

## 10. Physiological and Performance Effects of Sports Drinks Independent of Hydration

Intense exercise and physical training can cause not only dehydration and heat stroke, but also muscle and internal organ damage, immunodepression, systemic inflammation, oxidative stress, and fatigue, among other poor physical conditions [124]. Research to develop and evaluate functional foods and sports drinks that can help to ameliorate those conditions and promote early recovery is therefore being conducted [23,33,47,58,59,66].

For example, the hypotonic drink discussed earlier was found to inhibit interleukin 6 (IL-6) secretion during endurance exercise in male cyclists, indicating that hydration has the potential to reduce the inflammatory response during exercise [8,137,138,139]. Furthermore, in a study focusing on the menstrual cycle of women, leukocytes were observed to increase in the blood during exercise in a hot environment when the basal body temperature rose in the luteal phase, but that increase was suppressed by the consumption of the hypotonic drink [139]. Other inflammation-related factors such as IL-6 and leukocyte activation markers were also elevated, but no effect of the beverage was observed, suggesting that sex differences and exercise conditions might have influenced the results [140,141]. Another study that compared consumption of a hypotonic drink containing high-branched cyclic dextrin, a polymer of glucose and high GER [142] with consumption of an isotonic drink containing 5% glucose of the same calorie content during endurance races observed that urinary IL-8, IL-10, and IL-12p40 were reduced, suggesting that the hypotonic drink and/or the glucose polymer might reduce systemic inflammation and immunodepression [143].

Oral rehydration solution might promote the recovery of cerebral and fundus blood flow during intense exercise and might also promote early recovery of intravascular dehydration and dynamic visual acuity [47,144]. However, no effects on systemic inflammation and oxidative stress were observed with post-exercise fluid intake [47]. In any case, the type of beverage and timing of the intake were also considered important factors for effectiveness [145].

## 11. The Possibility of Changing the Composition of Sports Drinks

The findings for functional ingredients should be interpreted more cautiously than those for conventional carbohydrate–electrolyte beverages. Many studies on amino acids, bovine colostrum, ketone-related strategies, phytochemicals, caffeine, and other ergogenic or recovery-oriented ingredients are limited by small sample sizes, heterogeneous exercise protocols, differences in training status, and variable outcome measures. Therefore, although these ingredients may have physiological plausibility and potential practical value, their efficacy should not be generalized across all athletes or exercise settings. Future studies should compare acute and longer-term effects, include both supportive and non-supportive outcomes, and evaluate whether observed biomarker changes translate into meaningful performance or recovery benefits.

Intense exercise increases blood levels of lactic acid, free fatty acids and ketone bodies, resulting in blood acidification (acidosis) [146]. Alkaline water increases the body’s buffering capacity in maintaining acid–base equilibrium and might be useful in ameliorating exercise-induced metabolic acidosis [147,148]. In a study that combined alkaline water with glutamine, which has a protective effect on the intestinal tract and an ameliorative effect on immunodepression [149,150], α-amylase activity and testosterone concentration in saliva were observed to increase after boxing practice in what was considered to be a synergistic effect because beverage constituents are also known to have anti-inflammatory effects [151]. Testosterone is important in skeletal muscle anabolism [152,153,154,155,156,157,158,159], but is also known to decline in overtraining syndrome [160], and the foregoing change to the solvent and ingredients in sports drinks might be of use in the development of beverages that better support conditioning [161,162,163,164].

In addition to glutamine, leucine, isoleucine, and valine are collectively referred to as branched-chain amino acids (BCAAs), which are metabolized in skeletal muscle to provide energy; all are highly absorbable essential amino acids whose blood levels increase 15 min after oral intake [165]. In fact, BCAA-containing beverages have been found to be effective in ameliorating exercise-induced muscle damage and reducing muscle pain and fatigue [166]. Moreover, continuous intake of BCAAs leads to more efficient use of BCAAs as an energy source for performance and recovery [166]. They also suppress the production of lactic acid and might not only enhance endurance exercise capacity but also muscle mass and strength, although the magnitude of these effects may be context-dependent and evidence for performance enhancement remains inconsistent across exercise modalities and training backgrounds [167,168]. Milk is rich in BCAAs [165], and whey protein hydrolysates are also effective for muscle glycogen restorage after exercise [66].

Especially, bovine colostrum is the first milk, which includes growth and immune factors for the newborn, has been applied to sports nutrition for conditioning and performance [169,170,171]. Indeed, cyclists administered over a 9-week period were associated with an increased serum concentration of soluble tumor necrosis factor receptor 1, amelioration of post-exercise decreases in cytotoxic T-cells and serum immunoglobulin G_2_ concentration, and a reduced incidence of upper respiratory illness symptoms [172]. Bovine colostrum was later revealed to stimulate the release of interferon γ, IL-2, and IL-10, and to inhibit the early release of tumor necrosis factor, IL-4, and IL-6 in peripheral blood mononuclear cells, suggesting a potential mechanism for the benefits found to be associated with immunonutrition supports [173]. These findings are supported by limited evidence derived primarily from small trials, and further well-controlled studies are needed to confirm the practical relevance of bovine colostrum supplementation across diverse athletic populations and exercise contexts.

Similarly, hyperimmunized milk, obtained from cows vaccinated against specific pathogens (26 antigens including *Escherichia coli*, *Salmonella*, and *Staphylococcus aureus*), contains multiplicity of antibodies against pathogens and is associated with anti-inflammatory effects that protect intestinal function [174]. We investigated whether hyperimmunized milk as compared with usual milk protects against exercise-induced inflammation and organ injury in male runners [175]. The results suggested that urine specific gravity and urine osmolality decreased and that the urine concentrating ability in the kidney dropped after participants had run an all-out time trial of 3000 m [98]. However, urine concentrating ability was better maintained in the group treated with hyperimmunized milk, making those participants less prone to dehydration [175]. In addition, we demonstrated that eight weeks of hyperimmunized milk administration suppressed the elevation of I-FABP (a marker of intestinal injury) and inflammatory cytokines after all-out running, and that hyperimmunized milk intake can ameliorate exercise-induced intestinal damage and systemic inflammation [175]. In any case, frequent consumption of small boluses of a protein–carbohydrate beverage after exercise cessation is recommended to athletes to minimize gastrointestinal burden, support clearance and turnover of damaged epithelial and skeletal muscle tissue, and aid fluid retention [23,126,176,177].

Ketone diets and ketone drinks have also received considerable attention [178,179,180]. Long-term continuation of a ketone diet has been verified to induce keto-adaptation, improving endurance by allowing skeletal muscle to preferentially use ketone bodies and lipids as an energy source during exercise [181]. A sufficient supply of essential amino acids, in addition to supplying muscle glycogen, helps to improve and speed healing of injured muscle fibers [182]. Given those observations, a ketone diet that provides adequate protein at approximately 15% of daily calories can ameliorate amino acid deficits in skeletal muscle fibers [181]. On the other hand, the low carbohydrate content of this diet hampers muscle glycogen regeneration. As a result, the ketone bodies are rarely employed during high-intensity activity [182,183]. The evidence for ketone supplementation strategies therefore remains context-dependent, and the practical relevance for high-intensity sport performance is limited.

Drinkability and taste are also important aspects of sports drinks [184]. Many athletes dislike sweetened beverages and consume tea in sports settings [185]. Tea contains catechins and other compounds with anti-inflammatory, antioxidant, and antibacterial effects that also affect sports performance [186,187]. The effects of beverages with added catechins and carbohydrates (glucose and fructose) on exercise performance, systemic inflammation, and oxidative stress in athletes were therefore examined in cyclists [188]. No effects on those measures were observed but decreases in the blood lymphocyte counts and testosterone concentrations after exercise were ameliorated, which could be useful for athlete conditioning [187,188]. Notably, the drink used in the study contained caffeine at 6 mg per kilogram body weight; however, no effect was observed, at least on the sprint performance of the cyclists.

Similarly, glucosinolate-rich formulations, including sulforaphane-derived supplements, have attracted interest as phytochemical strategies to attenuate exercise-induced oxidative stress and support recovery. Some studies have reported beneficial effects, including protection against oxidative stress and improved adaptation to intense training, as well as attenuation of muscle soreness and damage following eccentric exercise [189,190]. However, a recent double-blind randomized crossover study found no significant effect of broccoli-derived glucoraphanin supplementation on recovery from eccentric muscle damage [191]. These contrasting findings indicate that findings are not consistent across studies, and that the efficacy of glucosinolate-derived compounds for exercise recovery remains context-dependent and not yet conclusive. Factors such as dose, bioavailability, intervention duration, exercise model, and timing of intake may all contribute to the variability in observed outcomes.

There is growing interest in research on caffeine as an ergogenic aid—a substance that enhances athletic performance—added to sports drinks [192,193]. Caffeine exerts its ergogenic effects primarily through central mechanisms, including antagonism of adenosine receptors in the brain, which attenuates perceived effort and fatigue; peripheral effects, including enhanced muscle contractility and altered substrate utilization, have also been reported, although their relative contribution may depend on dose and exercise context. Although caffeine is not currently classified as a prohibited substance, it is included in the World Anti-Doping Agency (WADA) Monitoring Program, indicating that its use should be carefully considered in competitive sports settings. With respect to recovery, caffeine intake in combination with carbohydrates has been reported to markedly enhance post-exercise muscle glycogen resynthesis [194]. In addition, caffeine ingestion has been associated with increased post-exercise IL-10 concentrations, suggesting a potential role in modulating post-exercise inflammatory responses and immune function [195]. While these findings indicate possible benefits of caffeine-containing beverages for post-exercise recovery, their practical relevance may depend on factors such as dosage, timing, individual responsiveness, and sport-specific regulatory considerations. However, these ergogenic benefits are not universally observed across all athletic populations and sport modalities; a randomized crossover study in basketball players demonstrated that pre-exercise caffeine supplementation did not consistently improve fatigue management outcomes, highlighting that applicability to real-world settings is limited and context-specific judgment is warranted [196]. Accordingly, caution and context-specific judgment are warranted when incorporating caffeine or other ergogenic aids into sports drinks.

In recent years, menthol has also been gaining attention. When menthol is consumed in combination with gels or cold/ice-filled slurry drinks, or when it is added to these products, it may promote a sensation of “coolness” during exercise [197,198]. The internal application of menthol is potentially ergogenic when performing endurance activities by ameliorating athlete’s perception of heat stress by reliably improving thermal sensation [198,199].

Despite the growing interest in these functional ingredients, their efficacy remains context-dependent and often supported by limited or heterogeneous evidence. In many cases, observed mechanistic changes do not consistently translate into meaningful improvements in performance, recovery, or health outcomes. Therefore, cautious interpretation is necessary, particularly when extrapolating findings to broader athletic populations.

## 12. Population-Specific Considerations and Research Gaps

Population-specific factors should also be considered when interpreting the efficacy and safety of sports drinks. Elite athletes may require individualized strategies based on sweat rate, training phase, competition rules, gastrointestinal tolerance, and recovery demands, whereas recreationally active individuals may benefit more from simple hydration guidance and avoidance of unnecessary sugar intake outside exercise settings. In adolescent athletes, sports drink use intersects with nutrition education, dental health, and long-term dietary behavior; however, evidence regarding efficacy, appropriate biomarkers, and safe intake patterns in this population remains limited. Recent work has begun to address this gap: a crossover study evaluating dairy-based beverages, including milk and kefir, for post-exercise recovery in young female athletes found evidence suggesting potential recovery benefits, while also highlighting the need for further investigation in this population [200,201]. The efficacy of various beverage types, the appropriate selection of outcome measures, and the establishment of safe intake recommendations for adolescent athletes remain important research priorities. In master or aging athletes, altered thirst perception, reduced voluntary fluid intake, hydration regulation, anabolic resistance, and slower recovery kinetics may substantially modify the response to carbohydrate–electrolyte or protein-containing beverages; direct evidence evaluating beverage strategies specifically in this population remains sparse, and extrapolation from younger athlete cohorts should be made with caution. Future studies should therefore examine whether beverage composition, timing, and outcome measures need to be adapted according to age, sex, training status, and health background.

## 13. Conclusions

Although this review incorporates evidence from established literature on traditional hydration-oriented sports drinks, it particularly focuses on recent studies examining sports drink formulations intended to provide benefits beyond hydration. The conclusions drawn in this review should be interpreted with consideration of several limitations, including heterogeneity among studies, relatively small sample sizes in some intervention trials, and variability in methodological rigor. Accordingly, the evidence summarized here is best regarded as illustrating emerging patterns and important areas for future investigation, rather than as definitive evidence of universal efficacy.

From this perspective, future research would benefit from explicitly differentiating acute responses from longer-term adaptations, accounting for hydration status and population-specific characteristics, and adopting study designs that better reflect real-world sporting environments. In particular, well-controlled longitudinal studies, the use of standardized and context-appropriate outcome measures, and ecologically valid protocols are needed to more clearly define when, how, and for whom specific sports drink strategies may be most effective.

## Data Availability

No new data were created or analyzed in this study. Data sharing is not applicable to this article.

## Abbreviations

GER	gastric emptying rate
I-FABP	intestine-type fatty acid-binding protein
L-FABP	liver-type fatty acid-binding protein
IL	interleukin
BCAA	branched-chain amino acid
WADA	world anti-doping agency
